# Trade-Offs of Flowering and Maturity Synchronisation for Pineapple Quality

**DOI:** 10.1371/journal.pone.0143290

**Published:** 2015-11-23

**Authors:** V. Nicodème Fassinou Hotegni, Willemien J. M. Lommen, Euloge K. Agbossou, Paul C. Struik

**Affiliations:** 1 Centre for Crop Systems Analysis, Wageningen University, Wageningen, the Netherlands; 2 Faculté des Sciences Agronomiques, Université d’Abomey Calavi, Cotonou, Benin; Mediterranean Agronomic Institute at Chania, GREECE

## Abstract

In the pineapple sector of Benin, poor fruit quality prevents pineapple producers to enter the European market. We investigated effects of common cultural practices, flowering and maturity synchronisation, (1) to quantify the trade-offs of flowering and maturity synchronisation for pineapple quality and the proportion of fruits exportable to European markets, and (2) to determine the effect of harvesting practice on quality attributes. Four on-farm experiments were conducted during three years using cultivars Sugarloaf and Smooth Cayenne. A split-split plot design was used in each experiment, with flowering induction practice as main factor (artificial or natural flowering induction), maturity induction practice as split factor (artificial or natural maturity induction) and harvesting practice as the split-split factor (farmers’ harvest practice or individual fruit harvesting at optimum maturity). Artificial flowering induction gave fruits with lower infructescence weight, higher ratio crown: infructescence length, and a lower proportion of fruits exportable to European markets than natural flowering induction. The costs of the improvements by natural flowering induction were huge: the longer durations from planting to flowering induction and harvesting, the higher number of harvestings of the fruits increasing the labour cost and the lower proportion of plants producing fruits compared with crops from artificially flowering-induced plants. Artificial maturity induction decreased the total soluble solids concentration in the fruits compared with natural maturity induction thus decreasing the proportion of fruits exportable to European markets, at a benefit of only a slightly shorter time from flowering induction to harvesting. Harvesting individual fruits at optimum maturity gave fruits with higher total soluble solids in naturally maturity induced fruits compared with the farmers’ harvest practice. Given the huge costs of natural flowering induction, options to use artificial flowering induction effectively for obtaining high fruit quality are discussed.

## Introduction

In most developing countries, primary producers often face difficulties to export their product to European countries due to poor quality [1–6]. This is certainly the case in the fresh pineapple chains in Benin where less than 2% of the pineapple is exported to Europe [7]. In Benin, primary producers fail to produce fresh pineapple meeting the quality criteria of other actors in the chains to local and regional markets [8]. Important quality attributes for these actors include the fruit weight, taste, juice pH (affecting the taste) and flesh translucency [8]. Also, the proportion of fresh pineapple exported to Europe is low due to the lack of compliance with the demands for quality of fruits set by the Codex Alimentarius [9]. Quality attributes considered in the Codex Alimentarius (including those called size attributes in the Codex) are fruit and infructescence weight, ratio crown length: infructescence length, total soluble solids (TSS), internal browning and flesh translucency. Fruit quality attributes can be affected by cultural practices and post-harvest practices [10–12]. Since pineapple fruit quality can hardly be improved by post harvest practices [4], this study concentrated on fruit quality issues in the field. Understanding the trade-offs of some common cultural practices (determining the fruit quality) for fruit quality would help to improve it.

In pineapple, the transition from the vegetative to the generative phase can take place in two ways. The first is by natural flowering induction (**NFI**), in which environmental stimuli are inducing flowering. These environmental stimuli can be: shortening of the day length [13], temperature dropping [14], reduction of daily sunshine hours due to cloudiness [15] and water deficit [16]. Natural flowering induction occurs in the presence of at least one of these factors [17] and when the plant has attained an appropriate size to capture and respond to enviromental stimuli [16]. The second and common way in pineapple cultivation is by artificial flowering induction (**AFI**) or “forcing”, which consists of applying growth regulators releasing acetylene or ethylene [17–20]. Artificial flowering induction (a) advances flowering, (b) improves uniformity of flowering, (c) makes the harvest moment predictable, and (d) makes harvesting more uniform [17,21,22]. However, AFI could probably constitute a source of poor fruit quality at harvest time when compared with NFI as all plants are induced to flower, no matter their size. Studies showed that there is an association between planting material weight at planting and the number of fruitlets involved in fruit weight (consequently the average fruit weight) at harvest on one hand [23] and between the plant weight at the moment of AFI and fruit weight at harvest on the other hand [18]. Thus, plants within a crop that are small at the moment of AFI would produce small fruits. To date, no research has reported the trade-offs of flowering induction practices for quality of pineapple fruits. We hypothesize that artificial flowering induction will lead to poorer fruit quality than NFI.

Not only flowering induction may account for poor quality at harvesting; maturity induction could be an additional source of poor quality. Fruit maturity can be induced in two ways: naturally or artificially. Natural maturity induction (**NMI**) is characterized by natural and gradual changes in the skin colour and in internal quality attributes such as TSS (an indicator of the sweetness of the pineapple juice) and juice pH [24]. From 12 to 4 weeks before harvesting time, TSS is low [25]. From 4 weeks before harvesting time, TSS increases until harvest time [25]. The pH starts to increase 2 weeks before harvesting time until harvesting time [26]. Artificial maturity induction (**AMI**) is achieved by applying an ethylene-releasing compound on the skin of the fruit. Such practice (a) hastens the change in the skin colour from green to yellow resulting in a uniformly yellow skin colour [27–29] and (b) concentrates the fruit harvesting. However, Hepton [18] argued that earlier AMI slows down both sugar accumulation and full cell expansion. Since the rate of the pineapple inflorescence development and growth varies among plants within a crop [24,30], we hypothesize that AMI to all fruits at the same moment will lead to overall poorer internal fruit quality attributes than NMI.

Harvesting time plays an important role in determining the final fruit quality [31]. Generally, fruits from artificially induced pineapple crops are harvested when 25% of the pineapple fruits in the field reaches harvesting maturity. That way of harvesting (**FH**, farmers’ harvesting practice) leads to harvesting fruits from the least and most advanced plants simultaneously and may reduce the average quality. We assume, as suggested by Muasya et al. [32] for crops grown from seed, that harvesting of individual pineapple fruits at their optimum harvesting time (**OH** practice) would allow fruits to develop their full potential before harvesting, which may yield a higher average quality compared with FH.

The objectives of this study were (1) to quantify the trade-offs of flowering and maturity synchronisation for pineapple quality and the proportion of fruits exportable to European markets and (2) to determine the effect of harvesting practice on fresh pineapple quality attributes. Four on-farm experiments were conducted during three years; plants were induced to flower naturally or artificially; fruit maturity was induced naturally or artificially and fruits were harvested according to the farmers harvest practice or the optimum harvest (for individual fruits) practice. Quality attributes and percentage of fruits exportable to Europe were assessed.

## Materials and Methods

### Ethics statement

We confirm that no specific permit was required for using the locations where the field trials were conducted. The locations were not protected in any way. The field study never involved endangered or protected species. Farmers provided permission to use their crops and lands and were financially compensated. The research was carried out by researchers living in the country and approved by local authorities and communities. The funders had no role in study design, data collection and analysis, decision to publish, or preparation of the manuscript.

### Experimental sites

**Fig 1:**
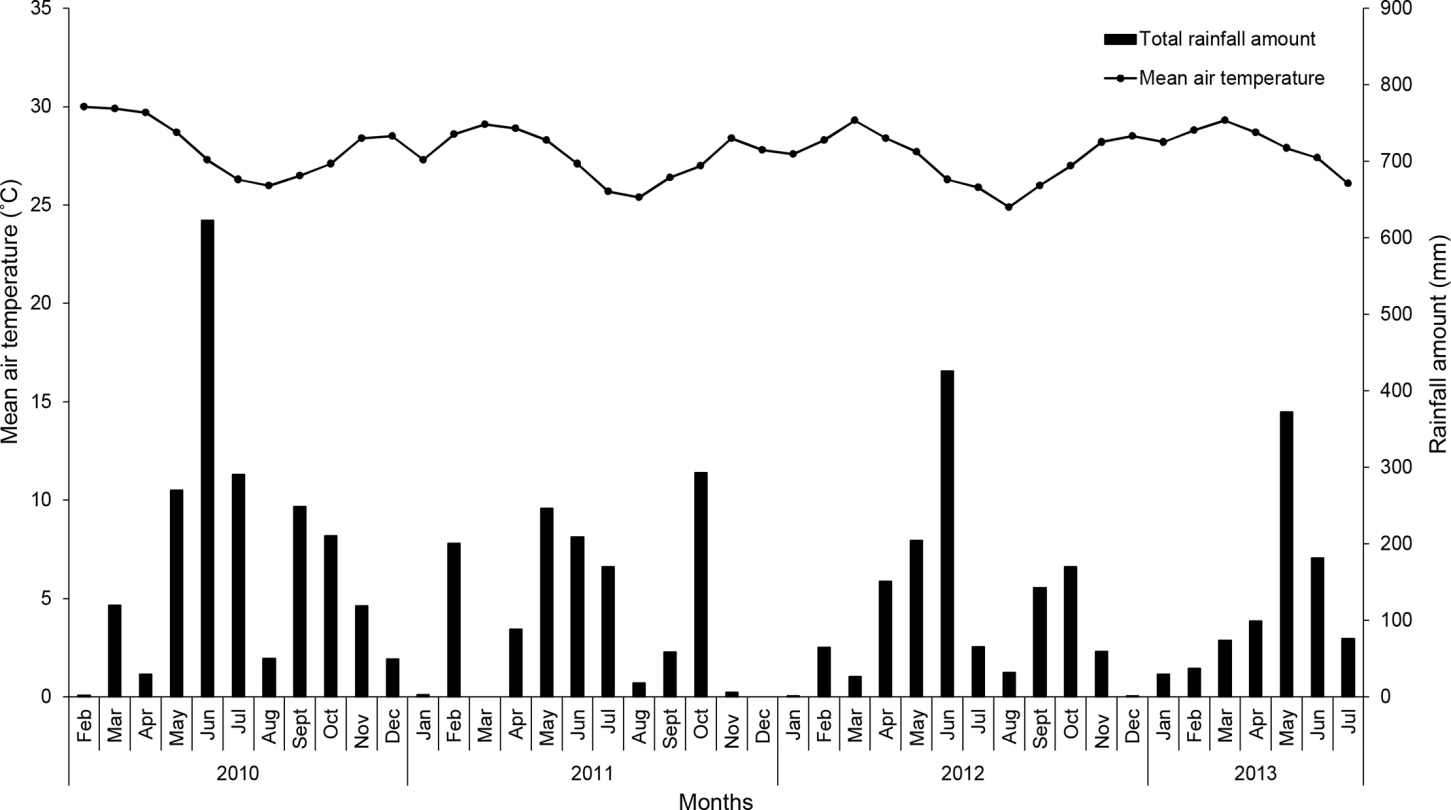
Variation in mean air temperature and monthly rainfall during the experimentation period (February 2010 to July 2013).

Four on-farm experiments were carried out on commercial pineapple fields in the Atlantic department in the south of Benin between February 2010 and July 2013. The pineapple cultivars used were the cultivar locally known as Sugarloaf -but possibly the same as cv. Pérola- in Experiments 1 and 2, and cv. Smooth Cayenne in Experiments 3 and 4. The experimental sites were selected on fields of different producers based on (a) the age of their pineapple crop being close to the common artificial flowering induction time and (b) whether they cropped their pineapple following the common practices as described by Fassinou Hotegni et al. [22]. Information on the fields and cultural practices until artificial flowering induction time is provided in Table 1. Mean air temperature and total rainfall amount during the experimentation period are provided in Fig 1. Experiments 1 and 2 were carried out from February 2010 to June 2013 and July 2010 to June 2013 respectively. Experiments 3 and 4 were carried out from April 2011 to July 2013 and May 2011 to June 2013, respectively.

**Table 1:** Field information and cultural practices in the four experiments with cvs Sugarloaf or Smooth Cayenne.

Field information and cultural practices	Cv. Sugarloaf		Cv. Smooth Cayenne	
	Experiment 1	Experiment 2	Experiment 3	Experiment 4
Location	06°36'09.2"N and 02°16'31.6"E	06°37'26.4"N and 02°16'13.1"E	06°36'43.7"N and 02°19'55.1"E	06°36'44"N and 02°19'54.3"E
Municipality (district)	Zè (Tangbo Djevie)	Zè (Tangbo Djevie)	Abomey-Calavi (Zinvié)	Abomey-Calavi (Zinvié)
FAO soil group (common name in Benin)	Ferralsols (Ferralitic soil)	Ferralsols (Ferralitic soil)	Ferralsols (Ferralitic soil)	Ferralsols (Ferralitic soil)
Climate	Subequatorial	Subequatorial	Subequatorial	Subequatorial
Planting time^a^	February 2010	July 2010	April 2011	May 2011
Type of planting material used^a^	Slips	Slips	Hapas and suckers	Hapas and suckers
Planting material treatment before planting^a^	No treatment	No treatment	No treatment	No treatment
Planting arrangement	Flat beds of two alternating rows	Flat beds of two alternating rows	Flat beds of two alternating rows	Flat beds of two alternating rows
Plant spacing: BP^b^ × BR^c^/BDR^d^ (cm)	40 ×50/80	35 × 45/65	47 × 55/75	47 × 55/75
Plant density (plants/m^2^)	3.85	5.19	3.27	3.27
First Urea (46N) + NPK (10-20-20)^a^	7 MAP^e^ (18 September 2010)	2 MAP (15 September 2010)	3 MAP (20 July 2011)	2 MAP (17 July 2011)
*Application form*	Solid at the base of the plants	Solid at the base of the plants	Solid at the base of the plants	Solid at the base of the plants
*Dose per plant (g Urea + g NPK)*	6 + 3	6 + 3	5 + 4	5 + 4
Second Urea (46N) + NPK (10-20-20)	Not applied	Not applied	6 MAP (15 October 2011)	5 MAP (24 October 2011)
*Application form*	Not applied	Not applied	Solid at the base of the plants	Solid at the base of the plants
*Dose per plant (g Urea + g NPK)*	Not applied	Not applied	4 + 5	4 + 5
NPK (10-20-20) application	12 MAP (22 February 2011)	9 MAP (16 April 2011)	Not applied	Not applied
*Application form*	Solid	Solid	Not applied	Not applied
*Dose per plant (g NPK)*	7	7	Not applied	Not applied
K_2_SO_4_ (50–18) application	Not applied	Not applied	10 MAP (8 February 2012)	9 MAP (17 February 2012)
*Application form*	Not applied	Not applied	Solid at the base of the plants	Solid at the base of the plants
*Dose per plant (g K*_*2*_ *SO*_*4*_ *)*	Not applied	Not applied	7	7
Artificial flowering induction time	13 MAP (6 March 2011)	10 MAP (4 May 2011)	10 MAP (22 February 2012)	10 MAP (3 March 2012)
Weed control	Hand weeding	Hand weeding	Hand weeding	Hand weeding

^a^ Information gathered from pineapple producer (field owner).

^b^ BP, spacing between plants within a row.

^c^ BR, width between rows.

^d^ BDR, spacing between double rows.

^e^ MAP, months after planting.

### Design, treatments, induction and harvesting practices

#### Design and treatments

In each experiment a split-split-plot design was used with four replicated blocks and three factors; the *flowering induction practice* was the main factor and had two levels: AFI and NFI; the *fruit maturity induction practice* was the split factor and had two levels: AMI and NMI; the *harvesting practice* was the split-split factor and had two levels: FH and OH. A net plot consisted of 60 plants arranged in 6 rows of 10 plants each. The net plots were surrounded by two guard rows and two guard plants within rows.

#### Flowering induction practice

In the AFI plots, plants were artificially induced between 10 and 13 months after planting (Table 1) using carbide of calcium (CaC_2_) (calcium carbide was only used to induce flowering, not to induce fruit maturity), a compound producing acetylene when it reacts with water. Following farmers’ practices for artificial flower induction, 50 ml of a solution containing 10 g/l and 15 g/l of CaC_2_ for Sugarloaf and Smooth Cayenne respectively, was applied into the centre of the leaf rosette of each plant. This application was carried out once in cv. Sugarloaf and three times, with an interval of three days, in cv. Smooth Cayenne.

In the NFI plots, environmental factors were the stimuli for the plants. These plants were weekly checked for inflorescence emergence (inflorescence emergence, also called red heart stage, refers to the stage at which the inflorescence starts to become visible at the centre of the leaf rosette. At the red heart stage, the inflorescence is surrounded by reddish short leaves at the base of the inflorescence). The date of inflorescence emergence was recorded and from that, the induction date was computed by subtracting 34 days; it is well known in Benin that the period between flowering induction and inflorescence emergence does not vary (likely due to the narrow range of climatic conditions) and lasts 34 days (confirmed by the main author's own observations throughout several growing seasons). In February 2013, i.e. three years and two and a half years after planting of cv. Sugarloaf in Experiments 1 and 2 respectively and two years after planting Smooth Cayenne in Experiments 3 and 4, there were still some plants in the NFI plots which had not flowered. The decision was made to discontinue checking the plants to be naturally induced for inflorescence emergence and induce the remaining plants artificially. Therefore, plants in the NFI plots which showed inflorescence emergence after February 2013 were excluded from the experiments.

#### Maturity induction practice

Following farmers’ practices, maturity of cv. Smooth Cayenne fruits was induced on individual fruits 143 days after flowering induction, by spraying 3.5 ml of a solution of 14 ml/l Ethephon (2-chloroethylphosphonic acid), a compound producing ethylene, on the skin of each fruit. This application was carried out twice with an interval of four days. In Benin, the practice of inducing maturity artificially is commonly applied in cv. Smooth Cayenne but not in cv. Sugarloaf [22]. On the artificially flowering-induced plants in Experiment 1, cv. Sugarloaf, since farmers’ criteria in determining the appropriate application time for Ethephon was not well known, Ethephon was applied once at 153 days after flower induction. This was found to be late because of occurrence of natural changes in skin colour before that moment. Through discussions with pineapple farmers and explorations of the pineapple fields in the experimental zone, we concluded that one application at 143 days after flower induction was appropriate for maturity induction in cv. Sugarloaf. Therefore, maturity induction was carried out on the naturally flowering-induced plants in Experiment 1 and on all AMI plots in Experiment 2, 143 days after flowering induction. This application was carried out once. In order to avoid carry-over effects of the Ethephon, a waterproof tarpaulin was used to cover the non-treated plots before AMI. The tarpaulins were removed immediately after treatment.

#### Harvesting practice

Pineapple fruits were hand-harvested. In the NMI plots, the FH practice was the moment when the skin colour had started to change from green to gold-yellow in at least 25% of the fruits in a net plot for the naturally maturity induced fruits; the OH practice was the moment when 25% of the skin of an individual fruit had changed from green to gold-yellow for the naturally maturity-induced fruits. In the AMI plots, 7 days after the application (second application in cv. Smooth Cayenne) of the Ethephon, all fruits changed to a fully yellow-orange colour at the same time. The FH and OH dates were therefore similar.

### Data collection

Four types of data were collected: data on the duration of the plant development phases on the individual plants within all plots; data on the number of harvestings of the fruits per plot; data on fruit quality at harvest on the individual plants within all plots; and percentage of exportable fruits per plot. Data on the plant development phases included the duration of the vegetative and generative phases and of the full period from planting to harvesting. The duration of the vegetative phase was defined as the time from planting to flowering induction. The duration of the generative phase was defined as the time from flowering induction to harvesting. Data on the number of harvestings of the fruits were collected per plot; it was defined as the number of harvestings of the fruits until the harvesting of all fruits (present) in a plot.

Data on quality attributes were collected on the fruits at harvest time. They included quality attributes important for actors in fresh pineapple chains to local and regional markets and those important for fruits to be exported to European markets. Data important for actors in fresh pineapple chains to local and regional markets included the fruit (infructescence + crown) weight, the TSS in the pineapple juice, juice pH (affecting the taste of the fruit) and flesh translucency. For fresh pineapple fruits to be exported to European markets, infructescence weight, fruit weight, ratio crown: infructescence length, TSS, flesh translucency and internal browning are important [9]. The percentage of exportable pineapple fruits was computed based on the quality criteria mentioned in the Codex Alimentarius [9] i.e. fruit weights ranging between 0.7 and 2.75 kg, the ratio crown: infructescence length between 0.5 and 1.5, a TSS of at least 12° Brix, no exceedingly translucent flesh and fruits free of internal browning (blackheart).

For determining the weight attributes, a scale was used. To determine the ratio crown: infructescence length, a ruler was used to measure each fruit component and the ratio was computed. To determine TSS, juice pH, percentage of translucent flesh and percentage of flesh showing blackheart symptoms, pineapples were cut longitudinally into two halves. A portion of the juice obtained from squeezing one half was used to determine TSS by a hand refractometer; another portion of that juice was used to determine the juice pH by a hand-held pH meter. The second fruit half was used to estimate visually the percentage of fruit with translucent flesh and internal browning following the methods of Paull and Reyes [33].

### Data analysis

Data were analysed using GenStat for Windows 16th Edition [34]. Percentage of naturally flowering-induced plants was calculated per month and the cumulative percentage was used to have an overview of the total percentage of naturally flowering-induced plants per plot under NFI.

A three-way ANOVA for a split-split-plot design was used to test the effects of the flowering induction, maturity induction and harvesting practice, and their interactions, on (a) average duration of the plant development phases, (b) number of harvestings of the fruits, (c) average fruit quality and (d) proportion of fruits meeting the minimum European markets criteria for pineapple fruit. Translucent flesh data were transformed using square root transformation $\left(\sqrt{x+0.5}\right)$ before analysis [35,36]. Data on proportion of fruits meeting the minimum European markets criteria for pineapple were transformed using arcsine transformation of the square root of the proportion before analysis [37]. Proportions equal to 0 or 1 were replaced by $\left(\frac{1}{4n}\right)$ and $\left[1-(\frac{1}{4n})\right]$ respectively, where n is the total number of fruits per net plot [37]. In case of interactions, means were separated using LSD. To determine which quality criteria did not meet the minimum European market criteria, different combinations of quality criteria were set and the percentage of non-exportable fruits for each combination of quality criteria was computed.

## Results

### Occurring of flowering and percentage of plants producing fruits at the end of the experiments

**Fig 2:**
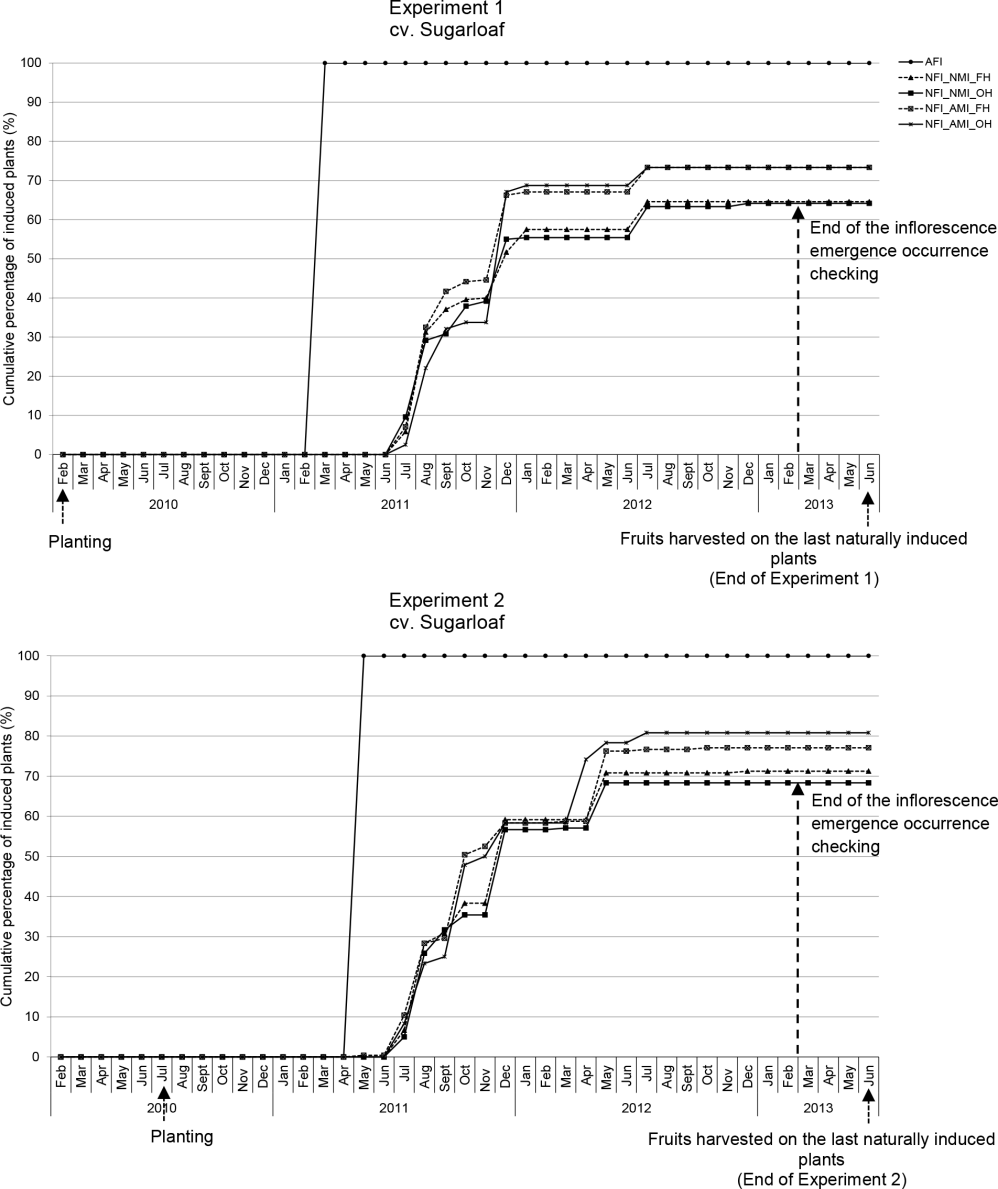
Cumulative percentage of flowering-induced plants in the different treatment combinations in cv. Sugarloaf, Experiments 1 and 2, until the harvesting of the fruits on the last naturally induced plants. AFI: Artificially flowering-induced plants (including all four AFI treatment combinations); NFI: Naturally flowering-induced plants. In February 2013, the decision was made to stop the regular checking of inflorescence emergence. AMI: Artificially maturity-induced fruits; NMI: Natural maturity-induced fruits. FH: Farmers’ harvesting practice; OH: Optimum harvest.

**Fig 3:**
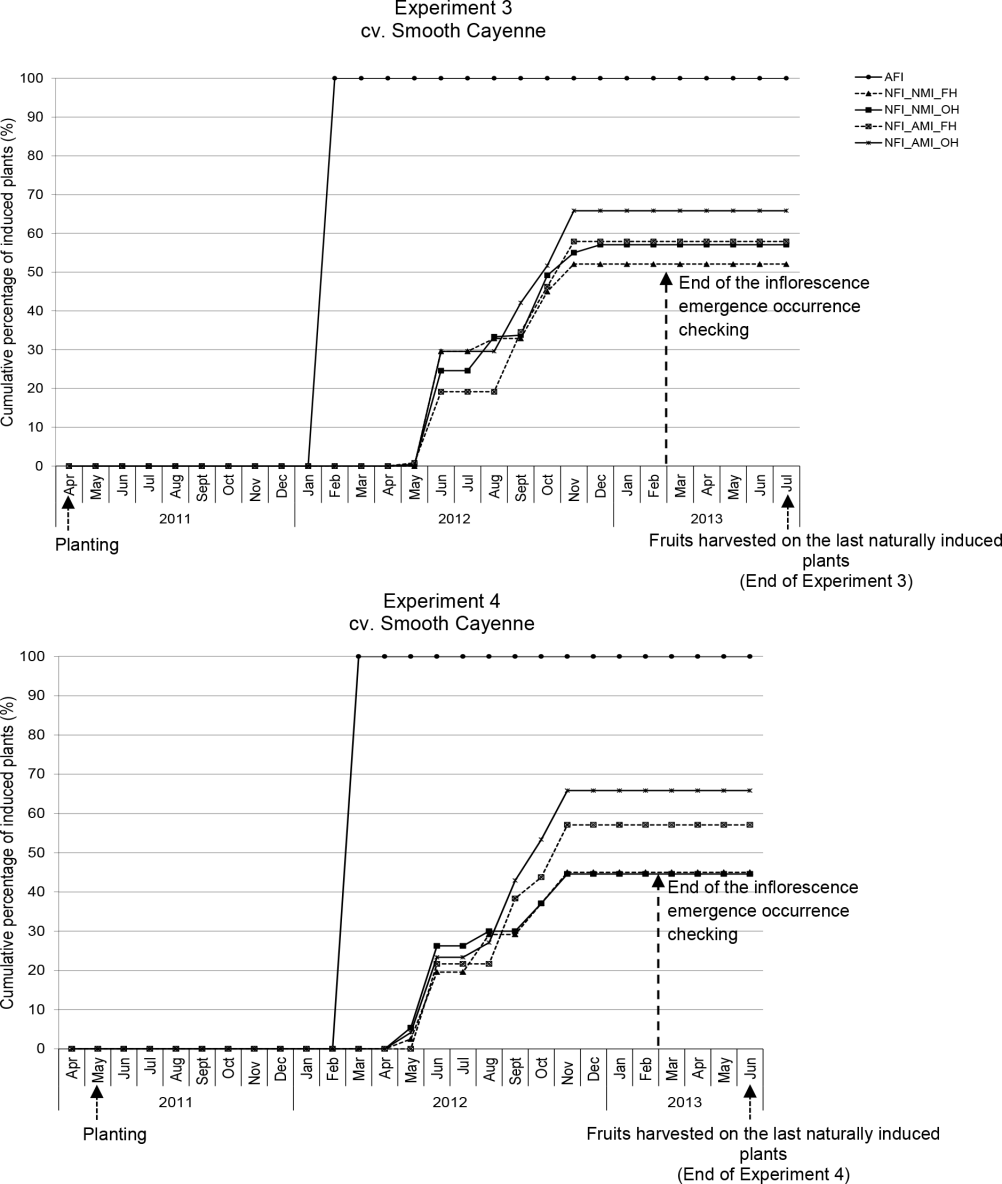
Cumulative percentage of flowering-induced plants in the different treatment combinations in cv. Smooth Cayenne, Experiments 3 and 4, until the harvesting of the fruits on the last naturally induced plants. AFI: Artificially flowering-induced plants (including all four AFI treatment combinations); NFI: Naturally flowering-induced plants. In February 2013, decision was made to stop the regular checking of inflorescence emergence. AMI: Artificially maturity-induced fruits; NMI: Naturally maturity-induced fruits. FH: Farmers’ harvesting practice; OH: Optimum harvest.

In all experiments, the AFI plants flowered uniformly after the carbide application (Figs 2 and 3). In the NFI plants, flowering occurred over a longer period with slight differences between the cultivars. In cv. Sugarloaf, plants were naturally induced mainly from July to January; in Experiment 2 some plants also were induced from March to May (Fig 2). The highest percentages of plants becoming naturally induced were recorded in August and December (Fig 2) in cv. Sugarloaf. In cv. Smooth Cayenne, plants were mainly induced naturally from May to November; some plants were induced in December (Fig 3). The highest percentages of plants becoming naturally induced were recorded in June and October in Experiment 3 and in June and November in Experiment 4 (Fig 3).

In all experiments, all AFI plants produced fruits. In the NFI treatments, the percentage of plants that had produced fruits at the end of the experiments ranged from 45% (108 out of 240 plants) to 81% (195 out of the 240 plants) (Figs 2 and 3).

### Duration of the plant development phases and number of harvestings of the fruits

#### Duration of the vegetative phase

**Fig 4:**
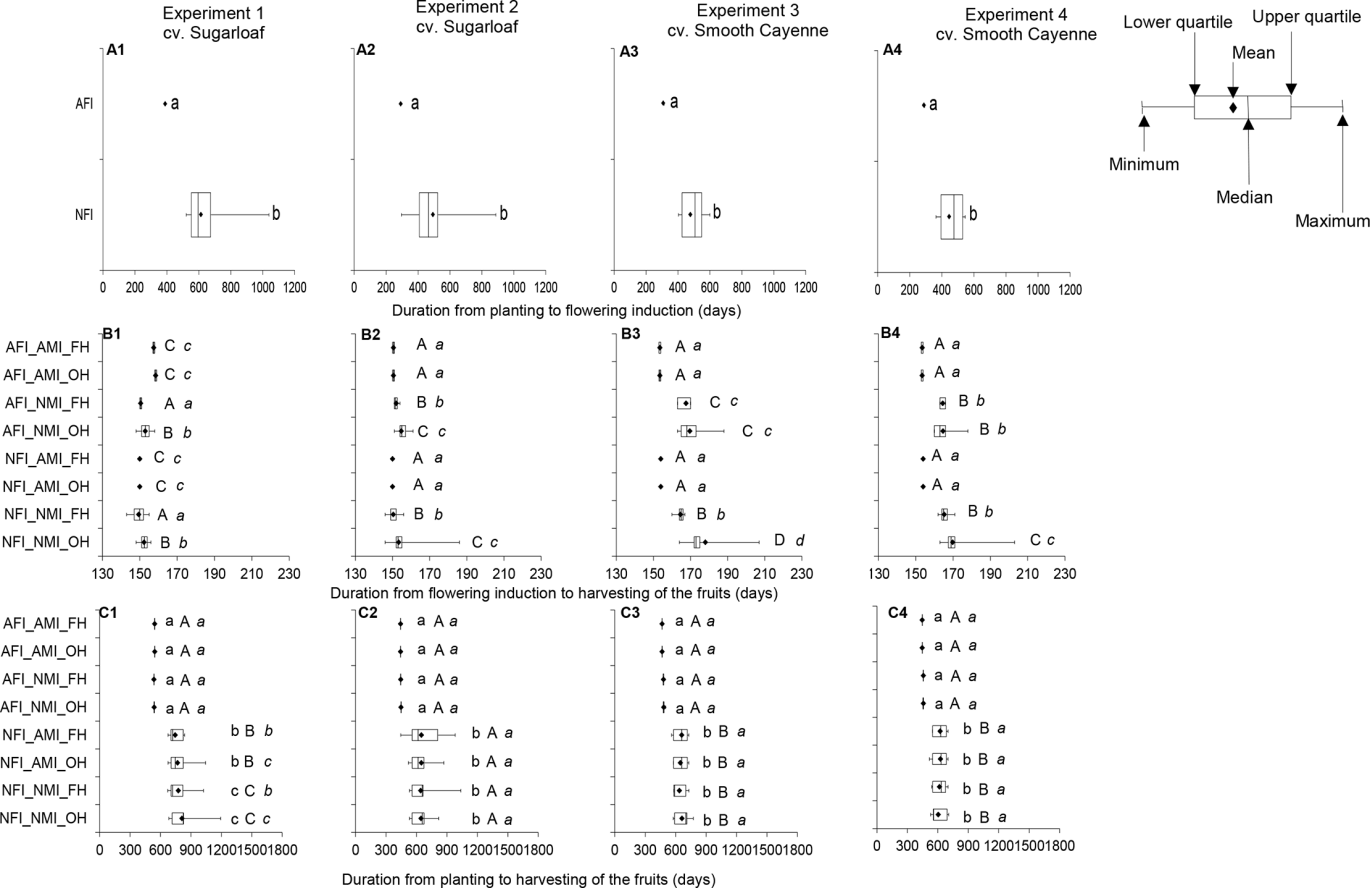
Boxplots with whiskers showing the effects of flowering induction practice on the average duration from planting to flowering induction (A) and the effects of flowering induction practice, maturity induction practice and harvesting practice on the average duration from flowering induction to harvesting of the fruits (B) and from planting to harvesting of the fruits (C) in cvs Sugarloaf (Experiments 1 and 2) and Smooth Cayenne (Experiments 3 and 4) and the distribution (maximum, minimum, quartiles, median) of each characteristic. AFI: Artificially flowering-induced plants; AMI: Artificially maturity-induced plants; NFI: Naturally flowering-induced plants; NMI: Naturally maturity-induced plants; FH: Farmers’ harvesting practice; OH: Optimum harvest. Next to the boxplots, similar *small* letters within a diagram indicate that differences in the duration between flowering induction practices are not significant based on the ANOVA results (consider P-values in bold in Table 2). Similar *capital* letters within a diagram indicate that differences in the duration between maturity induction practices are not significant based on the ANOVA results (consider P-values in bold in Table 2). Similar letters in *italic* within a diagram indicate that differences in the duration between harvesting practices are not significant based on the ANOVA results (consider P-values in bold in Table 2). All means are compared at LSD_0.05_ in case of interactions.

The effect of flowering induction practice on the average duration from planting to flowering induction was consistent in all experiments (Fig 4A; Table 2). Naturally flowering-induced plants had a longer duration of the vegetative phase than AFI plants. In NFI plants, the average duration from planting to flowering induction was at least 200 and 150 days longer than in AFI plants in cvs Sugarloaf and Smooth Cayenne, respectively. In the AFI treatments, all plants became induced to flower on the same date whereas in the NFI treatments, the time between the first and last induced plants varied from 164 to 535 days in cv. Sugarloaf and from 150 to 197 days in cv. Smooth Cayenne (Fig 4).

**Table 2:** P values of the F ratios from ANOVA for the effects of flowering induction practice, fruit maturity induction practice, harvesting practice and their interactions on time from planting to flowering induction, time from flowering induction to harvesting of the fruits, time from planting to harvesting of the fruits and on the number of harvestings of the fruits.

Variate/*Factor*	Cv. Sugarloaf		Cv. Smooth Cayenne	
	Expt 1	Expt 2	Expt 3	Expt 4
Duration from planting to flowering induction				
*Flowering induction practice*	**0.000** ***	**0.000** ***	**0.000** ***	**0.000** ***
Duration from flowering induction to harvesting of the fruits				
*Flowering induction practice (FIP)*	0.000 ***	0.038	0.072	0.051
*Maturity induction practice (MIP)*	0.000 ***	0.000 ***	0.000 ***	0.000 ***
*Harvesting practice (HP)*	0.000 ***	0.000 ***	0.000 ***	0.002 **
*FIP × MIP*	**0.000** ***	0.063	0.129	0.002 **
*FIP × HP*	0.561	0.825	0.004 **	0.003 **
*MIP × HP*	**0.000** ***	**0.000** ***	0.000 ***	0.002 **
*FIP × MIP × HP*	0.101	0.825	**0.004** **	**0.003** **
Duration from planting to harvesting of the fruits				
*Flowering induction practice (FIP)*	0.000 ***	**0.000** ***	0.000 ***	0.000 ***
*Maturity induction practice (MIP)*	0.027 *	0.796	0.006 **	0.833
*Harvesting practice (HP)*	0.001 **	0.784	0.623	0.654
*FIP × MIP*	**0.007** **	0.400	**0.001** **	**0.036** *
*FIP × HP*	**0.003** **	0.979	0.715	0.640
*MIP × HP*	0.349	0.782	0.191	0.432
*FIP × MIP × HP*	0.451	0.976	0.233	0.421
Number of harvestings of the fruits				
*Flowering induction practice (FIP)*	**0.000** ***	0.000 ***	0.000 ***	0.003 **
*Maturity induction practice (MIP)*	0.072	0.013 *	0.000 ***	0.000 ***
*Harvesting practice (HP)*	0.010 *	0.000 ***	0.000 ***	0.000 ***
*FIP × MIP*	0.465	0.837	0.080	0.001 **
*FIP × HP*	0.728	0.000 ***	0.036 *	0.039 *
*MIP × HP*	**0.000** ***	0.000 ***	0.000 ***	0.000 ***
*FIP × MIP × HP*	0.180	**0.000** ***	**0.012** *	**0.010** *

* Significant at the 0.05 probability level

** Significant at the 0.01 probability level

*** Significant at the 0.001 probability level

Values in **bold** indicate the P-value considered to establish the effect (main or interaction) of the flowering induction practice or the maturity induction practice or the harvesting practice.

#### Duration of the generative phase

Natural maturity induction led to a longer duration of the generative phase than AMI (Fig 4B) except in Experiment 1 (Fig 4B1) where the opposite was observed because maturity was artificially induced late as explained in the Materials and Methods section. In NMI treatments, the average duration of the generative phase was at least 1 day (value derived from Experiment 2 only, cv. Sugarloaf) longer in cv. Sugarloaf and 11 days longer in cv. Smooth Cayenne than in AMI treatments. In the AMI treatments, the difference between plants was 0 or 1 day whereas in the NMI treatments the difference between plants varied between 1 to 40 days in cv. Sugarloaf and 3 to 43 days in cv. Smooth Cayenne (Fig 4B).

In all experiments, harvesting practice did not affect the duration of the generative phase when AMI was applied (Fig 4B). When maturity was naturally induced, fruits harvested at OH showed a longer generative phase than those harvested at FH after all flowering induction treatments in cv. Sugarloaf and the NFI treatments in cv. Smooth Cayenne (Fig 4B). The generative phase of the fruits harvested at OH was 2 days and 1 day longer than that of fruits harvested at FH in cvs Sugarloaf and Smooth Cayenne, respectively.

#### Duration from planting to harvestings of the fruits

The effect of flowering induction practice on the duration from planting to harvesting of the fruits was consistent across experiments: NFI led to a longer duration than AFI (Fig 4C). Under NFI, the duration from planting to harvesting was between 196 and 274 days longer than that in AFI in the Sugarloaf experiments and between 146 and 192 days longer than that in AFI in the Smooth Cayenne experiments.

In Experiments 2 to 4, no significant effects of maturity induction practice on the duration from planting to harvesting were observed (Table 2). An effect was found only in Experiment 1 in the NFI plants where AMI led to shorter duration from planting to harvesting than NMI (Fig 4C1).

Effects of harvesting practice on the duration from planting to harvesting were found in Experiment 1 only and depended on the flowering induction practice (Table 2); under NFI treatment, the OH practice showed longer duration from planting to harvesting than the FH practice (Fig 4C1). In Experiments 2, 3 and 4, and the AFI treatments in Experiment 1, no significant effects of harvesting practice on the duration from planting to harvesting were observed (Table 2).

#### Number of harvestings of the fruits

**Fig 5:**
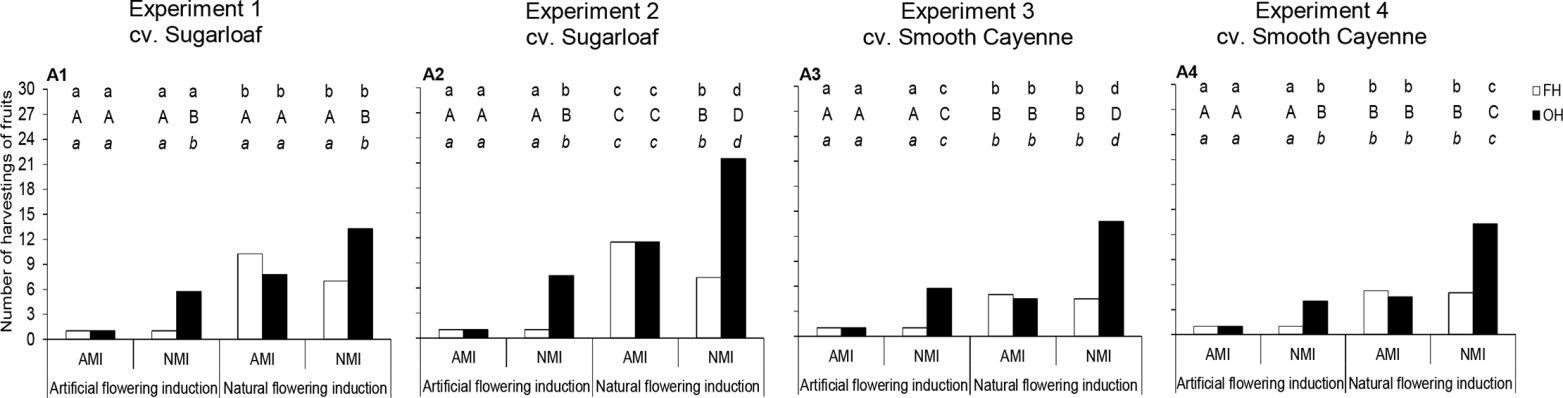
Effects of flowering and maturity induction practice and harvesting practice on the number of harvestings of the fruits in cvs Sugarloaf (Experiments 1 and 2) and Smooth Cayenne (Experiments 3 and 4). AMI: Artificially maturity-induced fruits; NMI: Naturally maturity-induced fruits; FH: Farmers’ harvesting practice; OH: Optimum harvest. Similar *small* letters at the top of each bar indicate that differences between means of the flowering induction treatments are not significant based on the ANOVA results (consider P-values in bold in Table 2). Similar *capital* letters at the top of each bar indicate that differences between means of the maturity induction treatments are not significant based on the ANOVA results (consider *P*-values in bold in Table 2). Similar *small* letters in *italic* at the top of each bar indicate that differences between means of the harvesting practice treatments are not significant based on the ANOVA results (consider *P*-values in bold in Table 2). In case of interactions all means are compared at LSD_0.05_.

The effects of flowering induction practice on the number of harvestings of the fruits were consistent across experiments. In all experiments, the number of harvestings in the NFI plots was 3 to 12 times and 2 to 6 times higher than that in the AFI plots in cvs Sugarloaf and Smooth Cayenne, respectively (Fig 5).

Effects of maturity induction practice on the number of harvestings of the fruits were also consistent across experiments. The maturity induction practice did not affect the number of harvestings in the treatments under AFI harvested at FH (Fig 5), but NMI increased the number of harvestings in the treatments under AFI harvested at OH as compared with AMI. When considering the treatments under NFI, NMI resulted in a comparable (Experiments 1, 3 and 4) or lower (Experiment 2) number of harvestings than AMI under FH, but more harvestings under OH (Fig 5).

Effects of harvesting practice on the number of harvestings of the fruits were also consistent across experiments. Harvesting practice did not significantly affect the number of harvestings when the fruits were artificially maturity-induced. When maturity was naturally induced, the number of harvestings was higher in the plots harvested at OH than that in the plots harvested at FH (Fig 5); in that case, harvesting at OH increased the number of harvestings by 3–8 and 2–6 times compared with the FH practice in cvs Sugarloaf and Smooth Cayenne respectively (Fig 5).

### Pineapple crop management practices and fruit quality at harvest

#### Effects of flowering induction practice on fruit quality

**Fig 6:**
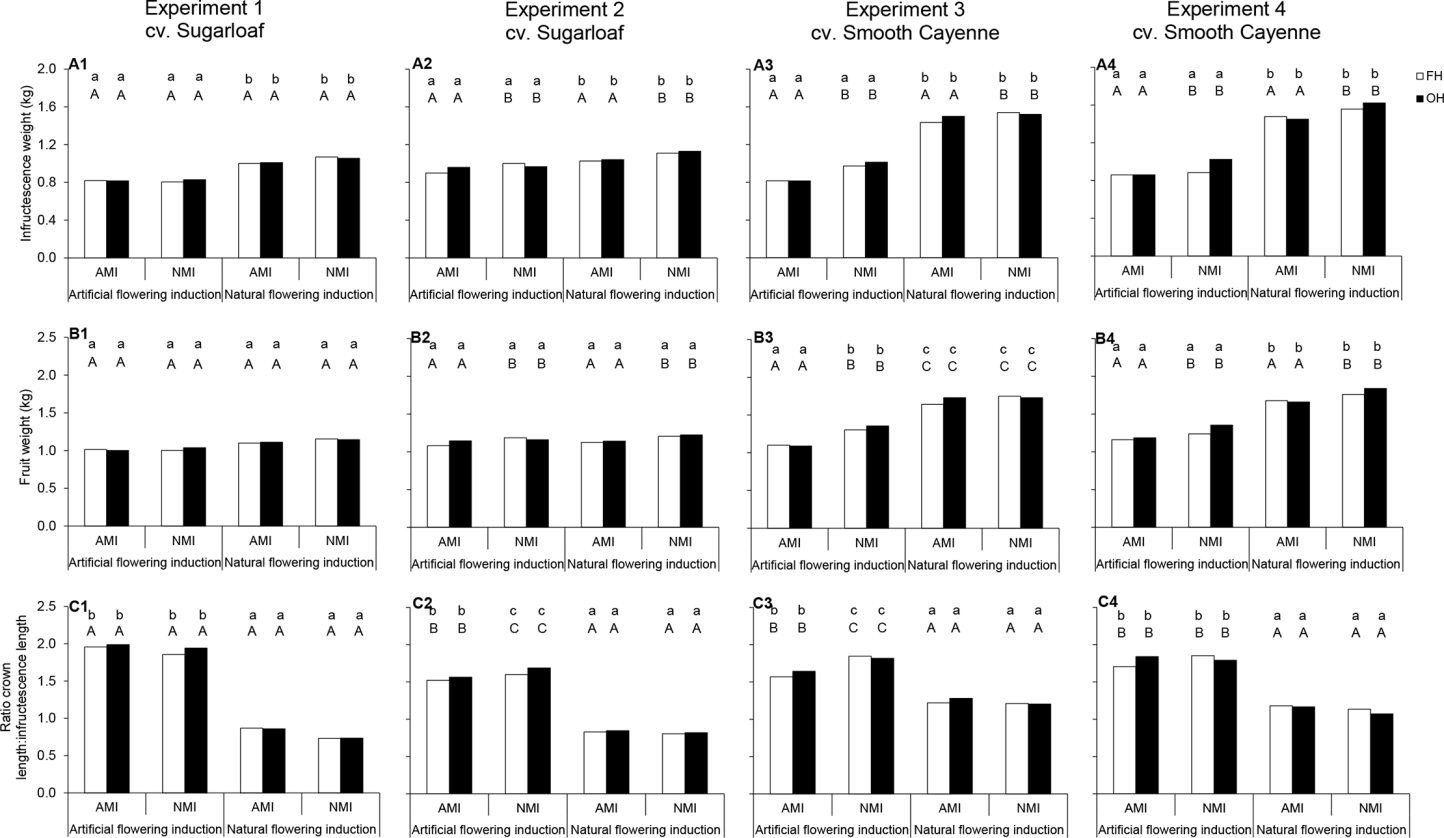
Effects of flowering and maturity induction practice and harvesting practice on the infructescence and fruit weights and ratio crown: infructescence length in cvs Sugarloaf (Experiments 1 and 2) and Smooth Cayenne (Experiments 3 and 4). AMI: Artificially maturity-induced fruits; NMI: Naturally maturity-induced fruits; FH: Farmers’ harvesting practice; OH: Optimum harvest. Similar *small* letters at the top of each bar indicate that differences between means in the flowering induction treatments are not significant based on the ANOVA results (consider P-values in bold in Table 3). Similar *capital* letters at the top of each bar indicate that differences between means in the maturity induction treatments are not significant based on the ANOVA results (consider P-values in bold in Table 3). In case of interactions all means are compared at LSD_0.05_.

The effect of flowering induction practice on the infructescence weight was consistent across experiments, but the effect on fruit weight was cultivar dependent (Fig 6; Table 3). Natural flowering induction resulted in fruits with higher infructescence weights (Fig 6A1 to 6A4) than AFI. Under NFI, there was an increase in the infructescence weights ranging from 9 to 33% and 50 to 84% compared with AFI in cvs Sugarloaf and Smooth Cayenne, respectively.

**Table 3:** P values of the F ratios from ANOVA for the effects of flowering induction practice, fruit maturity induction practice, harvesting practice and their interactions on average infructescence, crown and fruit weights and ratio crown: infructescence length in the two experiments per cultivar.

Fruit quality/*Factor*	Cv. Sugarloaf		Cv. Smooth Cayenne	
	Expt 1	Expt 2	Expt 3	Expt 4
Infructescence weight (kg)				
*Flowering induction practice (FIP)*	**0.008** **	**0.048** *	**0.000** ***	**0.000** ***
*Maturity induction practice (MIP)*	0.199	**0.033** *	**0.003** **	**0.004** **
*Harvesting practice (HP)*	0.742	0.510	0.546	0.261
*FIP × MIP*	0.192	0.568	0.058	0.529
*FIP × HP*	0.520	0.943	0.935	0.495
*MIP × HP*	0.936	0.354	0.734	0.152
*FIP × MIP × HP*	0.344	0.295	0.386	0.775
Fruit weight (kg)				
*Flowering induction practice (FIP)*	0.060	0.457	0.001 **	**0.000** ***
*Fruit maturity practice (MIP)*	0.179	**0.033** *	0.002 **	**0.003** **
*Harvesting practice (HP)*	0.560	0.439	0.528	0.243
*FIP × MIP*	0.426	0.676	**0.015** *	0.902
*FIP × HP*	0.698	0.957	0.878	0.666
*MIP × HP*	0.572	0.366	0.785	0.303
*FIP × MIP × HP*	0.179	0.351	0.272	0.990
Ratio crown: infructescence length				
*Flower induction practice (FIP)*	**0.000** ***	0.000 ***	0.000 ***	0.001 **
*Fruit maturity practice (FMP)*	0.079	0.160	0.006 **	0.447
*Harvesting practice (HP)*	0.250	0.053	0.446	0.968
*FIP × FMP*	0.589	**0.035** *	**0.000** ***	**0.004** **
*FIP × HP*	0.212	0.227	0.957	0.346
*FMP × HP*	0.493	0.592	0.216	0.138
*FIP × FMP × HP*	0.695	0.522	0.788	0.350

* Significant at the 0.05 probability level

** Significant at the 0.01 probability level

*** Significant at the 0.001 probability level

Values in **bold** indicate the P-value considered to establish the effect (main or interaction) of the flowering induction practice, the maturity induction practice or the harvesting practice.

In all experiments, the ratio crown: infructescence length was significantly lower in the fruits from NFI plants than in the fruits from AFI plants (Fig 6C1 to 6C4). The diminution in the ratio crown: infructescence lengths under NFI, ranged from 46 to 61% (cv. Sugarloaf) and 22 to 40% (cv. Smooth Cayenne).

**Fig 7:**
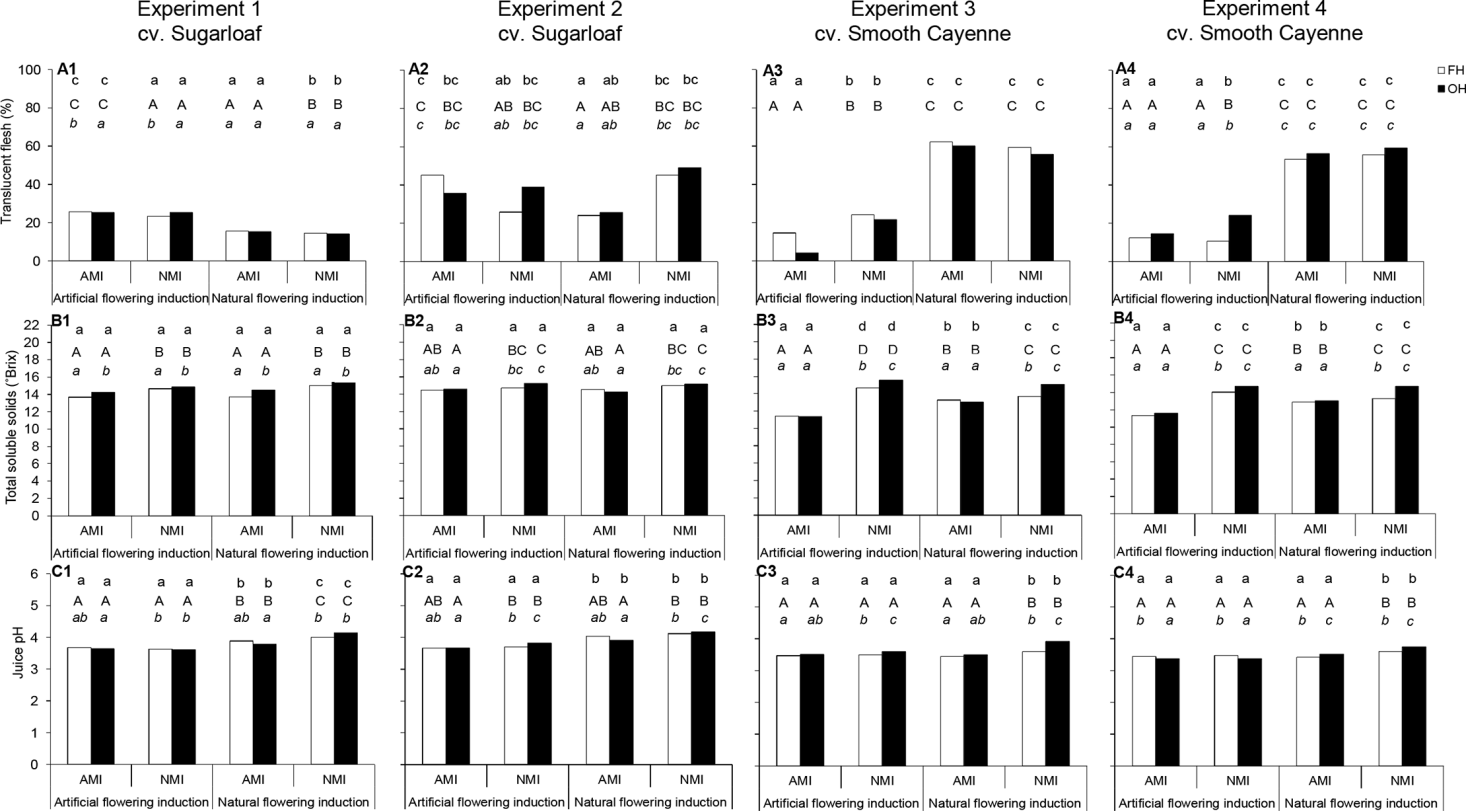
Effects of flowering induction practice, maturity induction practice and harvesting practice on percentage translucent flesh, total soluble solids and juice pH in cvs Sugarloaf (Experiments 1 and 2) and Smooth Cayenne (Experiments 3 and 4). AMI: Artificially maturity-induced fruits; NMI: Naturally maturity-induced fruits; FH: Farmers’ harvesting practice; OH: Optimum harvest. Similar *small* letters at the top of each bar indicate that differences between means in the flowering induction treatments are not significant based on the ANOVA results (consider P-values in bold in Table 4). Similar *capital* letters at the top of each bar indicate that differences between means in the maturity induction treatments are not significant based on the ANOVA results (consider P-values in bold in Table 4). Similar small letters in *italic* (when present) at the top of each bar indicate that differences between means in the harvesting practice treatments are not significant based on the ANOVA results (consider P-values in bold in Table 4). In case of interactions all means are compared at LSD_0.05_.

The effects of flowering induction practice on the percentage translucent flesh, TSS and juice pH depended on the cultivar (Fig 7; Table 4). In cv. Sugarloaf, the effect of the flowering induction practice on the percentage translucent flesh was not clear-cut across experiments and flowering induction practice had no significant effect on TSS (Table 3; Fig 7B1 and 7B2). Naturally flowering-induced Sugarloaf plants produced fruits with higher juice pH than AFI plants (Fig 7C1 and 7C2); the increase in juice pH ranged from 4 to 14%. In cv. Smooth Cayenne, NFI plants produced fruits with higher translucency than AFI plants (Fig 7A3 and 7A4); the percentage translucent flesh increased by more than 100% compared with AFI. The effects of flowering induction practice on TSS were consistent across Smooth Cayenne experiments under AMI treatments, where NFI plants gave fruits with higher TSS than AFI plants (Fig 7B3 and 7B4). Under the NMI treatments, the effects of flowering induction practice on TSS were not consistent. The effects of flowering induction practice on the juice pH were consistent across Smooth Cayenne experiments; flowering induction practice did not affect the juice pH under AMI treatments whereas under NMI treatments, NFI increased the juice pH (Fig 7C3 and 7C4). Internal browning was not observed in any fruit.

**Table 4:** P values of the F ratios from ANOVA for the effects of flowering induction practice, fruit maturity induction practice, harvesting practice and their interactions on average percentage translucent flesh, total soluble solids and juice pH in the two experiments per cultivar.

Fruit quality/*Factor*	Cv. Sugarloaf		Cv. Smooth Cayenne	
	Expt 1	Expt 2	Expt 3	Expt 4
Percentage translucent flesh				
*Flowering induction practice (FIP)*	0.028 *	0.285	0.000 ***	0.002 **
*Maturity induction practice (MIP)*	0.009 **	0.331	0.004 **	0.420
*Harvesting practice (HP)*	0.096	0.041 *	**0.038** *	0.017 *
*FIP × MIP*	**0.000** ***	0.005 **	**0.002** **	0.613
*FIP × HP*	**0.040** *	0.887	0.150	0.081
*MIP × HP*	0.471	0.000 ***	0.355	0.038 *
*FIP × MIP × HP*	0.458	**0.000** ***	0.072	**0.038** *
Total soluble solids				
*Flowering induction practice (FIP)*	0.145	0.942	0.039 *	0.019 *
*Maturity induction practice (MIP)*	**0.000** ***	0.012 *	0.000 ***	0.000 ***
*Harvesting practice (HP)*	**0.000** ***	0.139	0.011 *	0.001 **
*FIP × MIP*	0.135	0.477	**0.000** ***	**0.003** **
*FIP × HP*	0.260	0.057	0.693	0.291
*MIP × HP*	0.152	**0.024** *	**0.002** **	**0.015** *
*FIP × MIP × HP*	0.826	0.853	0.351	0.188
Juice pH				
*Flowering induction practice (FIP)*	0.009 **	**0.000** ***	0.048 *	0.010 *
*Maturity induction practice (MIP)*	0.047 *	0.000 ***	0.003 **	0.001 **
*Harvesting practice (HP)*	0.802	0.786	0.000 ***	0.167
*FIP × MIP*	**0.014** *	0.128	**0.018** *	**0.003** **
*FIP × HP*	0.490	0.162	0.026 *	**0.000** ***
*MIP × HP*	**0.036** *	**0.031** *	**0.004** **	0.985
*FIP × MIP × HP*	0.091	0.630	0.066	0.198

* Significant at the 0.05 probability level

** Significant at the 0.01 probability level

*** Significant at the 0.001 probability level

Values in **bold** indicate the P-value considered to establish the effect (main or interaction) of the flowering induction practice, the maturity induction practice or the harvesting practice.

#### Effects of maturity induction practice on fruit quality

Natural maturity induction gave fruits with higher infructescence weights than AMI (Fig 6A2 to 6A4), except in Experiment 1. When NMI occurred, there was an increase in the infructescence weights ranging from 8 to 11% and 1 to 24% compared with AMI in cvs Sugarloaf and Smooth Cayenne, respectively. In cv. Smooth Cayenne, the effect of maturity induction practice depended on the flowering induction practice (Table 3). Naturally maturity induced fruits had higher fruit weight than AMI fruits in AFI plants in Experiments 2, 3 and 4 and in NFI plants in Experiments 2 and 4 (Fig 6C2 to 6C4). In Experiment 1 and NFI plants in Experiment 3 there was no significant effect of maturity induction practice.

In Experiments 2, 3 and 4 the effects of maturity induction practice on the ratio crown: infructescence length depended on the flowering induction practice (Table 3). Maturity induction practice did not affect the ratio crown: infructescence length when the plants were naturally flowering-induced (Fig 6C1 to 6C4). In Experiments 2 and 3 results showed that NMI fruits from AFI plants had a higher ratio crown: infructescence length than AMI fruits (Fig 6C2 and 6C3); in Experiments 1 and 4 maturity induction practice did not significantly affect the ratio crown: infructescence length of the fruits originating from AFI plants (Fig 6C1 and 6C4).The effects of maturity induction practice on flesh translucency were not clear-cut in cv. Sugarloaf experiments; in cv. Smooth Cayenne experiments, maturity induction practice did not affect the flesh translucency of the fruits from the NFI plants (Fig 7A3 and 7A4). In AFI plants the effect of maturity induction practice on flesh translucency was not clear-cut (Fig 7A1 and 7A2). NMI fruits had generally a higher TSS than AMI fruits (Fig 7B1 to 7B4); when fruits were naturally maturity-induced, there was an increase in TSS compared with AMI fruits ranging from 2 to 10% and 3 to 37% in cvs Sugarloaf and Smooth Cayenne, respectively. Maturity induction practice in general did not affect the juice pH of the fruits from AFI plants, whereas NMI fruits had generally a higher juice pH than AMI fruits in NFI plants (Fig 7C1 to 7C4).

#### Effects of harvesting practice on fruit quality

In all experiments, harvesting practice had no significant effects on infructescence weight, fruit weight and the ratio crown: infructescence length (Table 3). Harvesting practice did not affect the percentage flesh translucency of the fruits from NFI plants (Fig 7A1 to 7A4). On the fruits from AFI plants, the same observations were made (Fig 7A2 to 7A4) except in Experiment 1 where harvesting of the fruits at OH gave fruits with a lower percentage translucent flesh than the FH practice (Fig 7A1). In all experiments except Experiment 1, the effect of harvesting practice on the TSS depended on the maturity induction practice (Table 4). Results were in general consistent and showed that NMI fruits harvested at OH had higher TSS than under the FH practice (Fig 7B1 to 7B4). For the AMI fruits, harvesting practice did not affect the TSS except in Experiment 1 where also AMI fruits harvested at OH showed higher TSS than fruits under the FH practice. In Experiments 1, 2 and 3, the effect of harvesting practice on the juice pH depended on the maturity induction practice. Harvesting practice did not significantly affect the juice pH of the AMI fruits in Experiments 1, 2 and 3; in the NMI fruits, the effect of harvesting practice on the juice pH was not clear-cut (Fig 7C1 to 7C4).

### Pineapple crop management practices and percentage of fruits exportable to European markets

**Fig 8:**
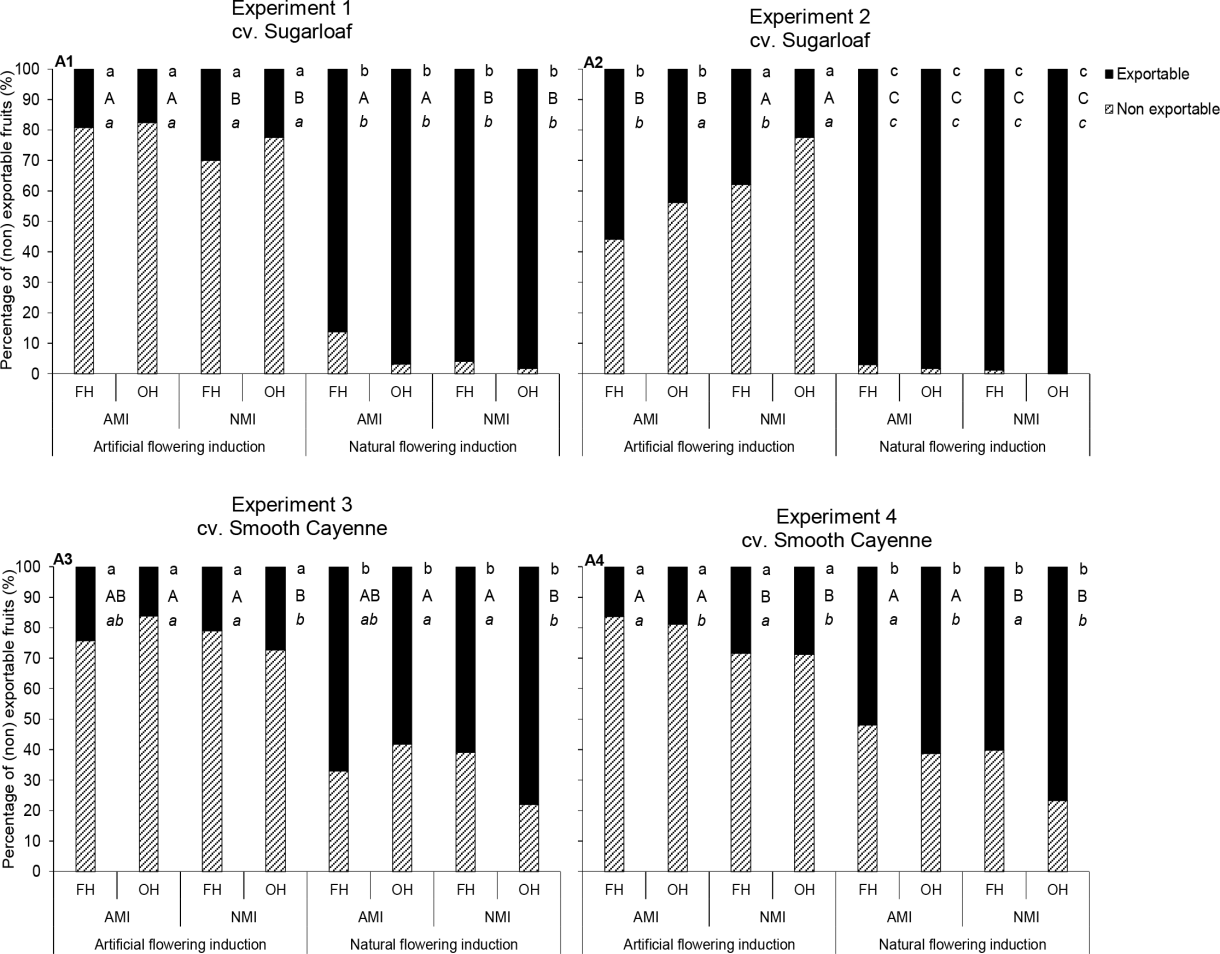
Effects of flowering induction practice, maturity induction practice and harvesting practice on the percentages of fruits that are exportable and non-exportable to European markets in cvs Sugarloaf (Experiments 1 and 2) and Smooth Cayenne (Experiments 3 and 4). AMI: Artificially maturity-induced fruits; NMI: Naturally maturity-induced fruits; FH: Farmers’ harvesting practice; OH: Optimum harvest. Similar *small* letters aligned close to the bars filled in black indicate that differences between the percentages of exportable fruits following the flowering induction practice are not significant based on the ANOVA results (consider P-values in bold in Table 5). Similar *capital* letters aligned close to the bars filled in black indicate that differences between the percentages of exportable fruits following the maturity induction practice are not significant based on the ANOVA results (consider P- values in bold in Table 5). Similar *small* letters in *italic* aligned close to the bars filled in black indicate that differences between the percentages of exportable fruits following the harvesting practice are not significant based on the ANOVA results (consider P-values in bold in Table 5). In case of interactions all means are compared at LSD_0.05_.

In none of the experiments fruits were found with exceedingly translucent flesh or internal browning. So, the percentage of fruits exportable to European markets was only affected by criteria for the fruit weight, the ratio crown: infructescence length and the TSS. In all experiments, flowering induction practice had significant (Table 5) and consistent effects on the percentage of fruits exportable to Europe (Fig 8). Naturally flowering-induced plants yielded a higher percentage exportable fruits than AFI plants (Fig 8A1 to 8A4); there was an increase in the percentage of exportable fruits compared with AFI of between 74 and 453% in cv. Sugarloaf and between 112 and 186% in cv. Smooth Cayenne.

**Table 5:** P values of the F ratios from ANOVA for the effects of flowering induction practice, fruit maturity practice, harvesting practice and their interactions on the percentage of fruits that are exportable to European markets in the two experiments per cultivar.

Factor	Cv. Sugarloaf		Cv. Smooth Cayenne	
	Expt 1	Expt 2	Expt 3	Expt 4
Flowering induction practice (FIP)	0.001 ***	0.000 ***	**0.000** ***	**0.003** **
Fruit maturity practice (MIP)	**0.003** **	0.011 *	0.050 *	**0.026** *
Harvesting practice (HP)	0.255	0.043 *	0.537	**0.037** *
FIP × MIP	0.911	**0.001** **	0.637	0.771
FIP × HP	**0.007** **	**0.002** **	0.328	0.118
MIP × HP	0.091	0.629	**0.013** *	0.866
FIP × MIP × HP	0.519	0.635	0.613	0.518

* Significant at the 0.05 probability level

** Significant at the 0.01 probability level

*** Significant at the 0.001 probability level

Values in **bold** indicate the P-value considered to establish the effect (main or interaction) of the flowering induction practice, the maturity induction practice or the harvesting practice.

The effect of maturity induction on the percentage of fruits exportable to Europe was not clear-cut in Experiments 2 and 3; in Experiments 1 and 4 NMI treatments gave a higher percentage exportable fruits than AMI treatments (Fig 8A1 and 8A4).

The effect of harvesting practice on the percentage of fruits exportable to Europe depended on the cultivar. In cv. Sugarloaf, harvesting practice did not affect the percentage of fruits exportable to Europe in fruits originating from NFI plants (Fig 8A1 and 8A2) whereas in fruits originating from AFI plants, the effect was not clear-cut (Fig 8A1 and 8A2). In the Smooth Cayenne experiments, the effect of harvesting practice on the percentage of fruits exportable to Europe was consistent. Harvestings of the NMI fruits at OH gave more exportable fruits than the FH practice (Fig 8A3 and 8A4); the increase in the percentage fruits exportable to Europe ranged between 14–30%. Harvesting of the AMI fruits at OH gave a similar percentage of exportable fruits as with FH practice in Experiment 3 (Fig 7A3); in Experiment 4, the percentage of exportable fruits at OH was higher than that from FH practice (Fig 7A4).

Our results also revealed that in cv. Sugarloaf, the ratio crown: infructescence length was the most limiting quality criterion because it had too high values (above 1.5) for a high percentage of fruits in the AFI plots (Table 6). In addition, small fruit weight limited the percentage of exportable fruits. In cv. Smooth Cayenne, there were two quality criteria limiting the proportion of exportable fruits: the ratio crown: infructescence length which was higher than 1.5 and the TSS which was less than 12°Brix (Table 7).

**Table 6:** Percentage of total fruits per treatment being non-exportable to European markets and falling within different set of quality criteria combinations in cv. Sugarloaf.

Fruit weight (kg)	Ratio crown: infructes-cence length	TSS (˚Brix)	Experiment 1								Experiment 2							
			Artificial flowering induction				Natural flowering induction				Artificial flowering induction				Natural flowering induction			
			AMI^a^		NMI^b^		AMI		NMI		AMI		NMI		AMI		NMI	
			FH^c^	OH^d^	FH	OH	FH	OH	FH	OH	FH	OH	FH	OH	FH	OH	FH	OH
**< 0.7** ^e^	[0.5–1.5]	≥ 12	0.00	0.84	0.83	0.00	0.00	0.64	0.00	0.00	0.00	0.00	0.42	0.00	2.35	0.00	0.00	0.00
**< 0.7**	**> 1.5**	**< 12**	0.42	1.25	0.42	0.00	0.00	0.00	0.00	0.00	0.00	0.00	0.00	0.00	0.00	0.00	0.00	0.00
**< 0.7**	**> 1.5**	≥ 12	13.75	12.92	17.50	11.67	0.66	0.00	0.00	0.00	5.83	3.75	3.33	3.75	0.58	1.12	0.00	0.00
[0.7–2.75]	**< 0.5**	≥ 12	0.00	0.00	0.00	0.00	1.32	0.64	2.80	1.72	0.00	0.00	0.00	0.00	0.00	0.00	1.29	0.00
[0.7–2.75]	[0.5–1.5]	**< 12**	2.91	0.42	0.00	0.00	6.62	0.00	1.40	0.00	0.00	0.42	0.00	0.00	0.00	0.00	0.00	0.00
[0.7–2.75]	**> 1.5**	**< 12**	2.50	0.83	0.00	0.00	0.00	0.00	0.00	0.00	1.25	1.25	0.00	0.00	0.00	0.00	0.00	0.00
[0.7–2.75]	**> 1.5**	≥ 12	**61.25** ^f^	**66.25**	**51.25**	**65.83**	5.30	1.91	0.00	0.00	**37.08**	**50.83**	**58.33**	**68.33**	0.00	0.56	0.00	0.00
**> 2.75**	[0.5–1.5]	≥ 12	0.00	0.00	0.00	0.00	0.00	0.00	0.00	0.00	0.00	0.00	0.00	8.75	0.00	0.00	0.00	0.00
**> 2.75**	**> 1.5**	≥ 12	0.00	0.00	0.00	0.00	0.00	0.00	0.00	0.00	0.00	0.00	0.00	1.67	0.00	0.00	0.00	0.00

^a^ Artificially maturity-induced fruits.

^b^ Naturally maturity-induced fruits.

^c^ FH: Farmers’ harvest practice.

^d^ Optimum harvest.

^e^ Quality criteria in **bold** refer to the quality criteria that do not respond to the quality requirement in the European markets.

^f^ Numbers in **bold** refer to where a huge number of pineapple fruits are not exportable to Europe.

**Table 7:** Percentage of total fruits per treatment being non-exportable to European markets and falling within different set of quality criteria combinations in cv. Smooth Cayenne.

Fruit weight (kg)	Ratio crown: infructes-cence length	TSS (˚Brix)	Experiment 3								Experiment 4							
			Artificial flowering induction				Natural flowering induction				Artificial flowering induction				Natural flowering induction			
			AMI^a^		NMI^b^		AMI		NMI		AMI		NMI		AMI		NMI	
			FH^c^	OH^d^	FH	OH	FH	OH	FH	OH	FH	OH	FH	OH	FH	OH	FH	OH
**< 0.7** ^e^	**< 0.5**	≥ 12	0.00	0.00	0.00	0.00	0.00	0.00	0.00	0.00	0.00	0.00	0.00	0.00	0.00	0.00	0.00	0.00
**< 0.7**	[0.5–1.5]	**< 12**	0.44	1.30	0.00	0.00	0.00	0.63	0.00	0.00	0.00	0.00	0.00	0.65	0.00	0.00	0.00	0.00
**< 0.7**	[0.5–1.5]	≥ 12	0.00	0.00	0.93	0.00	0.00	0.63	0.00	0.00	0.00	0.00	0.00	0.65	1.46	0.72	0.00	0.00
**< 0.7**	**> 1.5**	**< 12**	7.04	8.70	0.93	0.00	0.00	0.00	0.00	0.00	8.97	8.33	6.51	0.00	0.00	0.00	0.00	0.00
**< 0.7**	**> 1.5**	≥ 12	1.76	0.00	1.39	1.95	0.00	0.63	3.05	0.00	3.41	1.75	3.72	4.57	0.73	0.00	0.00	0.00
[0.7–2.75]	**< 0.5**	≥ 12	0.00	0.00	0.00	0.00	0.00	0.00	0.00	0.00	0.00	0.00	0.00	0.00	0.00	0.00	3.70	2.80
[0.7–2.75]	**< 0.5**	**< 12**	1.32	0.00	0.00	0.00	0.00	0.00	0.43	0.00	0.00	0.00	0.00	0.00	0.00	0.00	0.00	0.00
[0.7–2.75]	**< 0.5**	≥ 12	0.00	0.00	0.00	0.00	0.00	0.00	1.31	0.00	0.00	0.00	0.00	0.00	0.00	0.00	0.00	0.00
[0.7–2.75]	[0.5–1.5]	**< 12**	17.62	19.56	4.65	0.97	15.10	12.65	6.55	0.00	19.65	9.64	0.46	0.00	21.17	15.83	14.81	0.00
[0.7–2.75]	**> 1.5**	**< 12**	**32.15** ^f^	**38.69**	6.97	0.48	1.43	4.43	1.74	0.00	**34.18**	**43.42**	7.90	2.61	2.92	1.44	0.00	0.93
[0.7–2.75]	**> 1.5**	≥ 12	15.41	15.65	**64.18**	**69.26**	15.10	18.98	26.63	23.67	17.52	17.98	**53.02**	**62.74**	13.87	15.11	12.96	6.54
**> 2.75**	**< 0.5**	≥ 12	0.00	0.00	0.00	0.00	0.00	0.00	0.00	0.00	0.00	0.00	0.00	0.00	0.00	0.00	0.93	0.93
**> 2.75**	[0.5–1.5]	**< 12**	0.00	0.00	0.00	0.00	0.72	0.63	0.00	0.00	0.00	0.00	0.00	0.00	2.19	0.72	0.00	0.00
**> 2.75**	[0.5–1.5]	≥ 12	0.00	0.00	0.00	0.00	0.72	3.16	4.36	4.83	0.00	0.00	0.00	0.00	5.84	5.04	7.41	0.00
**> 2.75**	**> 1.5**	≥ 12	0.00	0.00	0.00	0.00	0.00	0.00	0.00	0.00	0.00	0.00	0.00	0.00	0.00	0.00	0.00	12.15

^a^ Artificially maturity-induced fruits.

^b^ Naturally maturity-induced fruits.

^c^ FH: Farmers’ harvest practice.

^d^ Optimum harvest.

^e^ Quality criteria in **bold** refer to the quality criteria that do not respond to the quality requirement in the European markets.

^f^ Numbers in **bold** refer to where a huge number of pineapple fruits are not exportable to Europe.

## Discussion

### Trade-offs and benefits of flowering synchronisation

**Fig 9:**
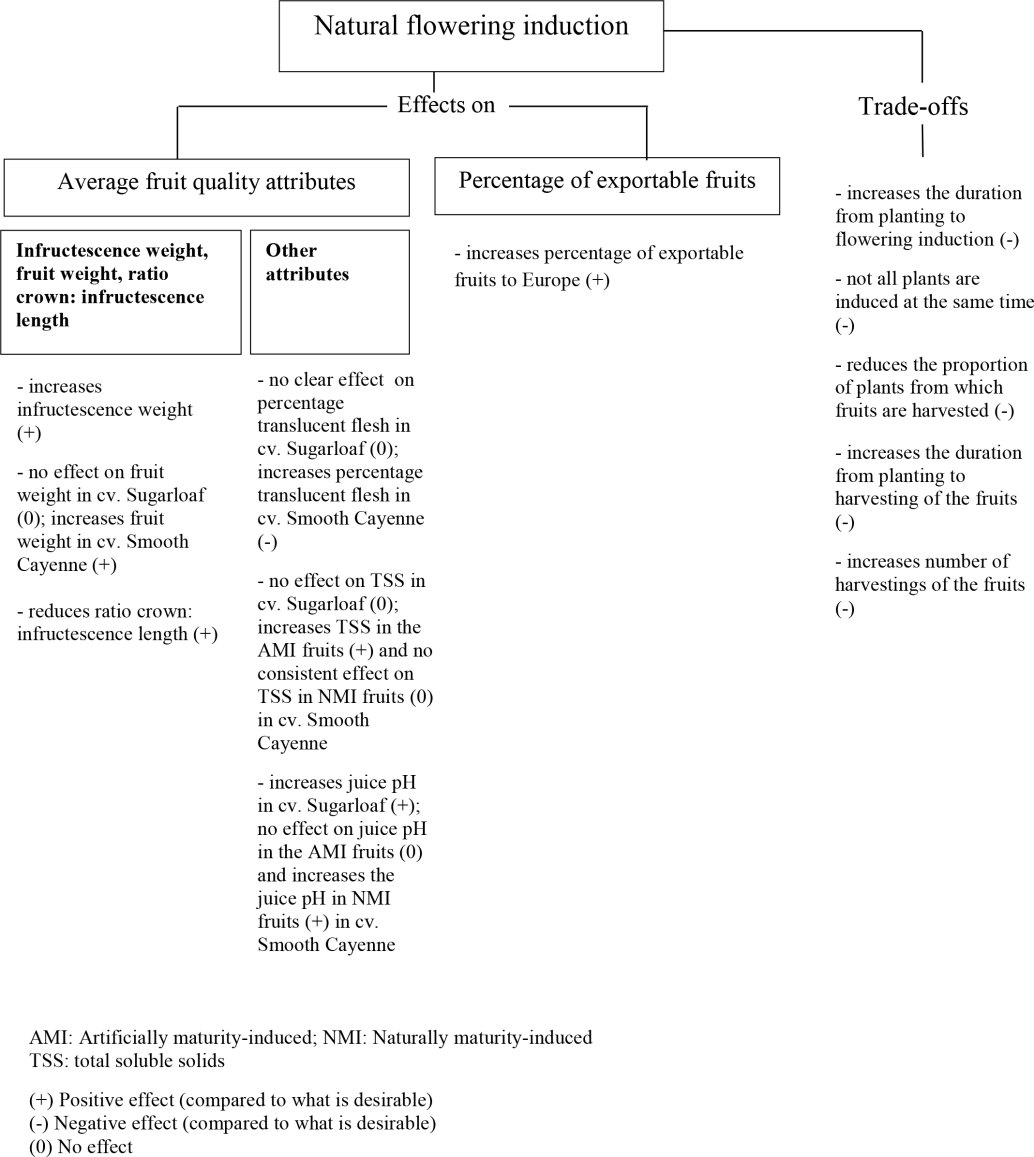
Effects and trade-offs of natural flowering induction vs. artificial flowering induction in a pineapple crop.

One of the objectives of this study was to quantify the trade-offs of flowering synchronisation for pineapple quality and proportion of exportable fruits. Our results clearly indicated that AFI produced lower fruit quality compared with NFI (Fig 9). Artificially flowering-induced plants gave fruits with lower infructescence weight (Fig 6A1 to 6A4) and a higher ratio crown: infructescence length when compared with NFI plants (Fig 6C1 to 6C4). Artificial flowering induction did not change the fruit weight in cv. Sugarloaf (Fig 6B1 and 6B2); in cv. Smooth Cayenne, AFI gave lower fruit weight than NFI (Fig 6B3 and 6B4). These effects of AFI on fruit weight and ratio crown: infructescence length, led to a reduction in the percentage of fruits exportable to European markets by more than 50% in the two cultivars compared with NFI (Fig 8). Thus in overall, NFI plants produced better average fruit quality than AFI plants. There was no financial cost for flowering induction of NFI plants, but the other costs (treated below) of achieving higher average fruit weight and lower ratio crown: infructescence length through NFI were very large.

First, in NFI, the time from planting to flowering induction was on average 200 and 150 days longer than that in the AFI plants in cvs Sugarloaf and Smooth Cayenne respectively. Such a longer time from planting to flowering induction in the NFI plants might allow them to reach a larger size [17] and become more vigorous at the moment of induction. Recent works by Fassinou Hotegni et al. [38] showed the existence of strong associations between the vigour of individual plants within a crop at (artificial) flowering induction and the infructescence and fruit weights and the ratio crown: infructescence length. Plants that are more developed at flowering induction are likely to produce heavier infructescences and lower ratio crown: infructescence length [38]. In the present study, due to the longer time from planting to flowering, NFI plants must be more vigorous at flower induction than AFI plants, and more assimilates may have been available at flowering induction time in NFI plants. Consequently, NFI plants were likely to produce fruits with heavier infructescence and shorter ratio crown: infructescence length (Fig 6).

Second, NFI plants were induced to flower over a long period of time and not at the same date as was the case in the AFI (Figs 2 and 3); there was a large time lag between the first NFI plants and the last NFI plants: 164–535 days and 150–197 days in cvs Sugarloaf and Smooth Cayenne, respectively (Fig 4). Most natural flowering inductions occurred during the coldest months (August and December) in cv. Sugarloaf and the wettest month (June) in cv. Smooth Cayenne (Figs 1–3), an important stimulus being the reduction of the intensity of solar radiation. Such more continuous flowering induction conditions of NFI plants might have played a role in achieving fruits with higher infructescence and fruit weights compared with AFI plants (Fig 6A1 to 6A4; Fig 6C1 to 6C4). This view is supported by the observations that NFI plants produced infructescences with more fruitlets called “eyes” than AFI plants (data not shown). In the case of tomato (*Solanum lycopersicum*) Adams et al. [39] found tomato plants exposed to low temperatures produced more flowers per truss than those exposed to relatively higher temperatures. In the case of citrus (*Citrus sinensis*), Moss [40] found that citrus plants exposed to low temperatures produced more flowers per inflorescence than those exposed to high temperatures.

Third, the average time from planting to harvesting of the NFI plants was 196–274 days longer than that of the AFI plants in cv. Sugarloaf and 146–192 days in cv. Smooth Cayenne (Fig 4). Allowing pineapple plants to flower naturally will thus oblige pineapple producers to keep their field under pineapple for a long period. The extra days could alternatively be used to grow cereals such as maize (*Zea mays*) that has a crop cycle of 75–90 days (early-maturing cultivars) in Benin [41] and/or legume crops such cowpea (*Vigna unguiculata*) that has a crop cycle of 90 days [42].

Fourth, under natural flowering induction practice, not all fruits produced by the NFI plants were harvested on the same day as was the case for AFI plants (Figs 4 and 5). This increases the number of harvestings of the fruits from NFI plots, which was 3 to 12 times and 2 to 6 times higher than that in the AFI plots in cvs Sugarloaf and Smooth Cayenne, respectively (Fig 5).

Fifth and last, the proportion of plants from which fruits were harvested ranged from 45–81% in the NFI treatments and was 100% in the AFI treatments (Figs 2 and 3). The percentage of NFI plants that produced fruits thus was lower than that of AFI plants. Even two years after planting some NFI plants could not flower and consequently could not produce fruits. This might be explained by huge differences in plant sensitivity within a crop to the natural flowering agents. The size and uniformity of the planting material might also play a role and non-flowering induced NFI plants could be those grown from smaller planting material which did not reach the appropriate size to capture natural flowering induction stimuli [24] or experienced too strong competition from surrounding plants (c.f. [43]).

All five reasons mentioned above, i.e. the increase in the number of days from planting to flowering induction, the non-synchronous flowering induction, the increase in the number of days from planting to harvesting, the high number of harvestings of the fruits and the decrease in the percentage plants that actually produced fruits are reasons that will jeopardize the acceptance of natural flowering induction practice by pineapple producers. Therefore, other options are needed while applying AFI in order to obtain a fruit quality closer to that obtainable under NFI.

#### Improvement options under artificial flowering induction practice

Earlier planting or planting heavier planting material with a narrow weight interval would help producers to obtain fruits with higher infructescence and fruit weights and with lower ratio crown: infructescence length when artificial flowering induction is applied [23]. Also, later artificial flowering induction based on the developmental status of the plants may help producers to obtain vigorous plants at the moment of flowering induction and consequently fruits with higher infructescence and fruit weights and with lower ratio crown: infructescence length closer to that obtained with natural flowering induction (cf. [18]). In this case, plants should be planted at appropriate planting density to avoid early resource competition among plants [43]. However, very late artificial flowering induction should be avoided since it may lead to an increase in competition for resources among and within plants. In that situation, AFI plants may produce lower average fruit since under competition for light and nutrients pineapple plants tend to produce fruits with low average fruit weight and length [43,44] and low sugar concentration [24]. In the case of cv. Smooth Cayenne where farmers have to induce three times (increasing the cost of artificial flowering induction), induction in the month during which the plants are more sensitive to natural flowering stimuli, i.e. in June, would improve the efficacy of the induction and may also improve fruit quality since it is well known that the quality of forcing agent also plays a role in fruit quality at harvest [45]. Farmers could probably also reduce the concentration of carbide of calcium solution or reduce the number of flowering inductions in that month.

Another improvement option could be the use of organic manure combined with fertiliser application which both will promote vegetative growth and improve infructescence and fruit weights as shown by Devadas and Kuriakose [46]. This will certainly increase the production cost but will increase plant vigour before artificial flowering induction.

#### Trade-offs and benefits of maturity synchronisation

**Fig 10:**
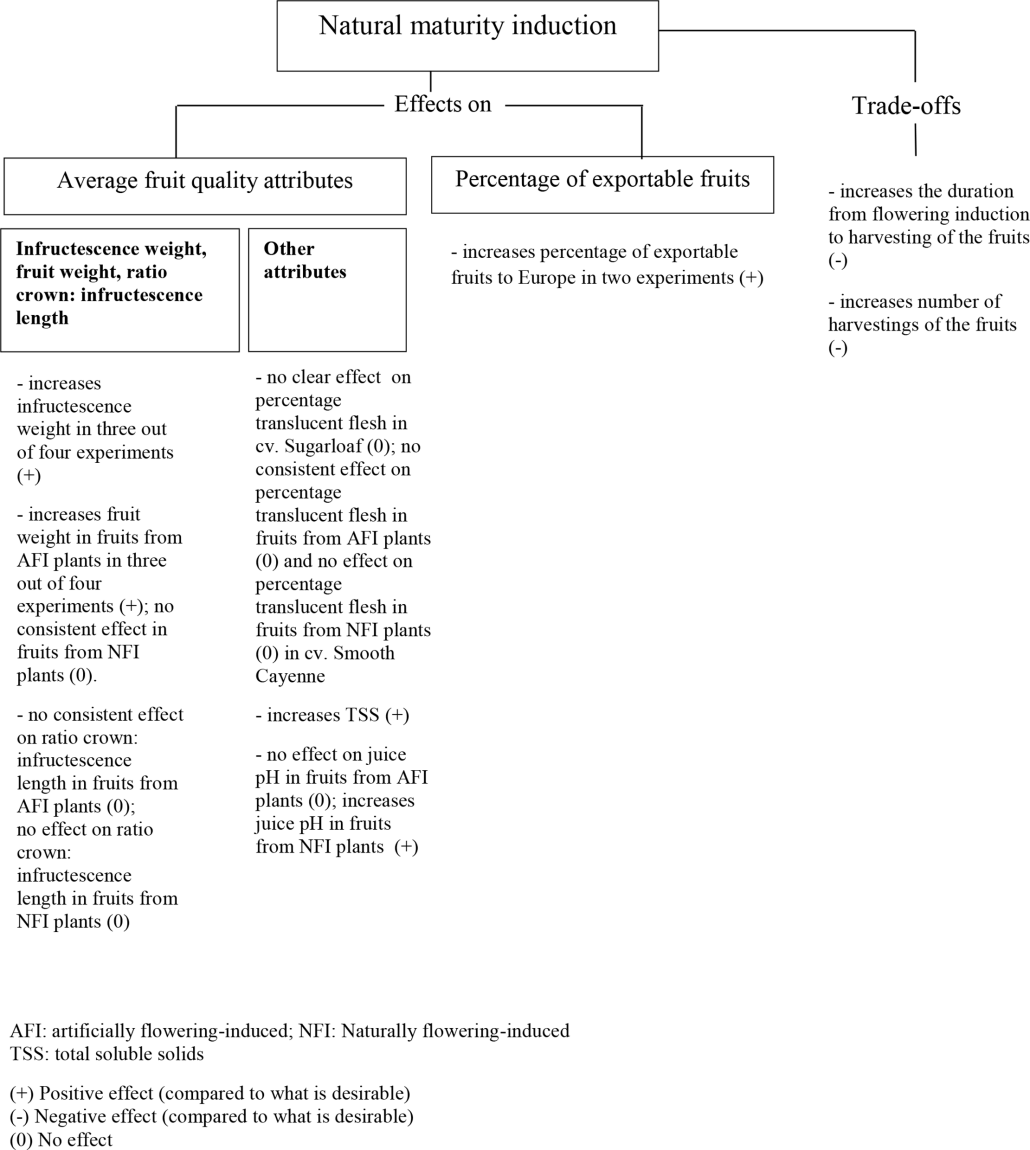
Effects and trade-offs of natural maturity induction vs. artificial maturity induction in a pineapple crop.

A second objective of this study was to quantify the trade-offs of maturity synchronisation for pineapple quality and proportion of fruits exportable to Europe (Fig 10). In Experiments 2, 3 and 4, AMI fruits presented lower infructescence weights than NMI fruits (Fig 6A2 to 6A4). The same was found for fruit weight except in Experiment 3 where fruits from NFI plants showed similar fruit weights no matter the maturity induction practice. In all experiments, NMI fruits were sweeter than AMI fruits (Fig 7B1 to 7B4). Artificial maturity induction led to a small decrease in the proportion of exportable fruits, mainly in Experiments 1 and 4 (Fig 8A1 and 8A4) when compared with NMI; this lower proportion of exportable fruits was a consequence of a lowering of the TSS by the AMI.

The positive effect of natural maturity induction on fruit weight (Fig 10) through the infructescence weight was not expected but can be explained. The infructescence growth follows a sigmoid curve with a slight increase during the last weeks before the harvesting time [47]. The increase of the infructescence weight during the last weeks is accompanied by flattening of the fruitlets at the skin of the fruits through further expansion of the fruitlets due to the increase in cell volume. When AMI was carried out, the degree of flattening in the shell slowed down (main author’s personal observation), suggesting a limited capacity of the infructescence to further increase in size. This conclusion is in line with Hepton [18] who argued that fruit weight increased less when AMI was carried out earlier. Reasons why the NMI gave sweeter fruits than AMI can be found in the increase in TSS, and especially the sucrose accumulation occurring during the last two weeks before harvesting [25]. Similar effects of NMI on TSS compared with AMI have thus far only been reported by Crochon et al. [28].

There were no financial costs for farmers for Ethephon application for maturity induction of NMI fruits; the other costs involved in obtaining NMI fruits were not as huge as those of NFI; they were related to the longer generative period and the higher number of harvestings (Fig 10). The period between flowering induction and harvest was 1 to 11 days longer in NMI than in AMI fruits. The number of harvestings of the fruits was only higher in the NMI treatments than AMI treatments when fruits were harvested at OH (Fig 5).

#### Effects of harvesting practice

**Fig 11:**
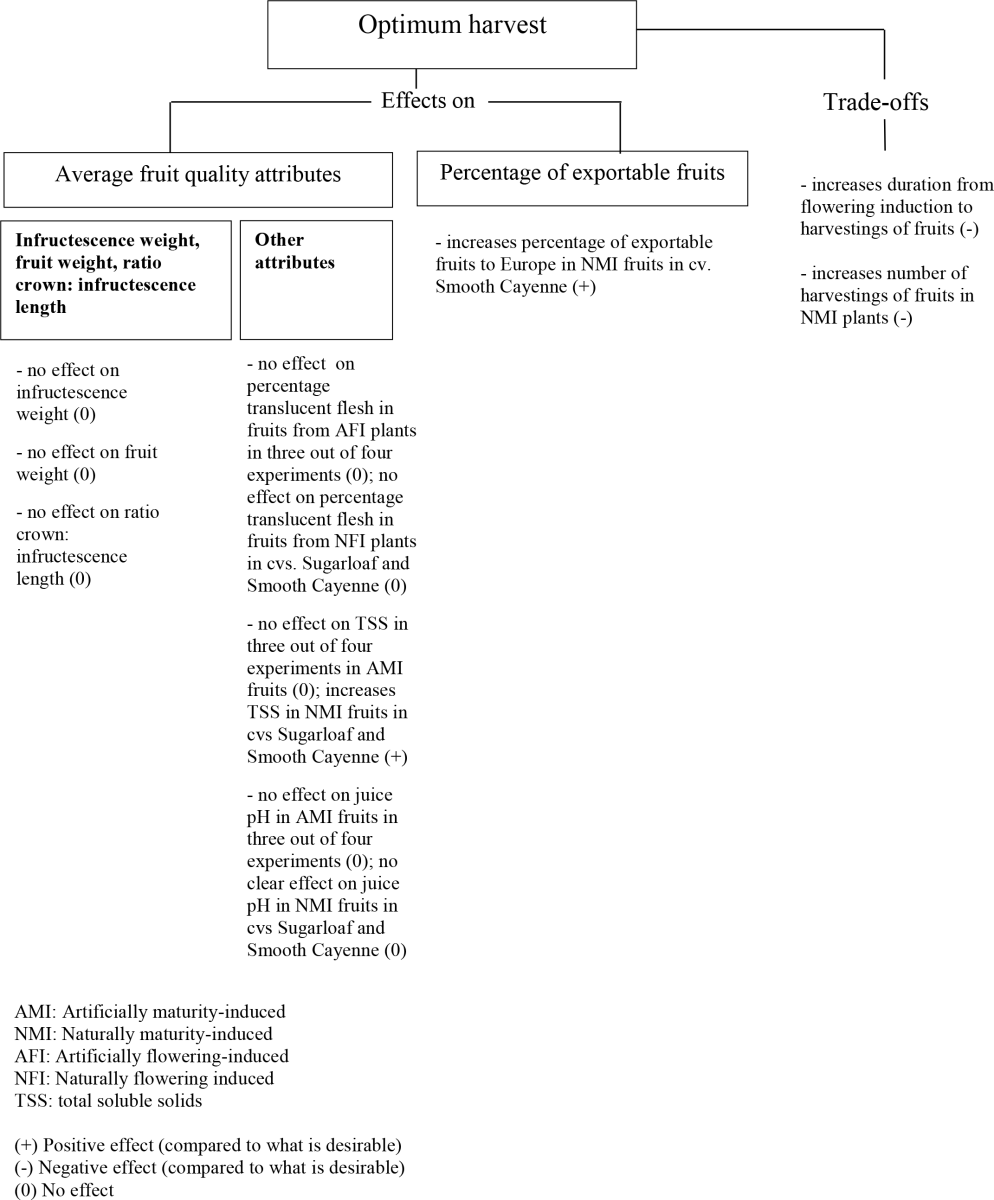
Effects and trade-offs of optimum harvest vs. farmers' harvest practice in a pineapple crop.

Our results indicated that harvesting practice had no significant effect on infructescence and fruit weights as well as on the ratio crown: infructescence length (Table 3; Figs 6 and 11). Harvesting practice in general did not affect the percentage translucent flesh (Fig 7A2 to 7A4). In Experiments 1, 3 and 4, NMI fruits harvested at OH had higher TSS than the FH practice (Fig 7B1, 7B3 and 7B4). This was not the case for the AMI fruits where harvesting practice had in general no effect on the TSS. Harvesting practice in general did not affect the juice pH of the AMI fruits (Fig 7C1, 7C2 and 7C3). When considering the percentage of fruits exportable to Europe, our results showed no effect of harvesting practice on the percentage of exportable fruits in cv. Sugarloaf under NFI treatments (Fig 8A1 and 8A2). In cv. Smooth Cayenne harvestings of the NMI fruits at OH increased the percentage of fruits exportable to Europe by 14–30% compared with the FH practice (Fig 8A3 and 8A4).

The reason why harvestings of the fruits at OH gave higher TSS than the FH practice under NMI is that first, fruits matured naturally and second they were harvested individually at their moment of 25% gold-yellow skin coloration. In these conditions the natural change in the TSS mainly the increase in the sucrose [26] was not disrupted by maturity induction or premature harvesting of a proportion of the fruits, but continued until harvesting the fruits at the optimum moment. This explains why the percentage of exportable fruits was higher in cv. Smooth Cayenne. In cv. Sugarloaf, the TSS was overall higher than in cv. Smooth Cayenne and was not a main export-limiting criterion. In the FH practice, since all fruits were harvested in one operation, the immature fruits or the fruits that did not reach their optimum harvesting time lowered the average TSS.

The extra costs of obtaining fruits with higher TSS at OH were two fold (Fig 11). First, harvestings of the fruits at OH increased the duration from flowering induction to harvestings of the fruits by at least 1 day in cv. Sugarloaf and 2 days in cv. Smooth Cayenne compared with the FH practice (Fig 4). Second, harvestings of the fruits under NMI treatments at OH increased the number of harvestings of the fruits by 3–8 and 2–6 times compared with the FH practice in cvs Sugarloaf and Smooth Cayenne respectively. Such increase in the number of harvestings of the fruits might increase the harvesting costs i.e. the labour costs because each time producers might need external help to harvest the fruits.

## Conclusions and Implications

Flowering and maturity synchronisation are common practices in pineapple cultivation. Our experiments showed that these practices contribute to poor fruit quality and to a low percentage of fruits that are exportable to European markets in the present pineapple production systems in Benin. Potentially, when crops were allowed to become naturally induced to flower, the average infructescence and fruit weights would become higher, the ratio crown: infructescence length would become reduced, and a higher percentage of fruits would be exportable to the European markets compared with crops receiving artificial flowering induction. However, the costs to gain these improvements in fruit quality attributes were huge: very long time from planting to flowering induction and from planting to harvesting, a high number of harvestings of the fruits and a low proportion of plants producing fruits compared with the crops from artificially flowering-induced plants.

Based on the huge costs of natural flowering induction, improvements while using artificial flowering induction become the best options to improve fruit quality. Options to use artificial flowering induction to obtain fruits with higher infructescence and fruit weights and lower ratio crown: infructescence length include cultural practices such as earlier planting or planting of heavy uniform planting material, later artificial flowering induction, and the use of organic manure combined with fertiliser application during the vegetative phase. Induction of cv. Smooth Cayenne in June could reduce flowering induction cost.

Experiments also showed that when crops were allowed to mature naturally, the fruits had higher TSS concentrations than when maturity was synchronized artificially, making a larger proportion of the Smooth Cayenne fruits exportable, whereas only a slightly longer time from flowering induction to harvesting of the fruits was needed to obtain this.

## Supporting Information

S1 File Experimental data Exp 1–4 Trade-offs Pineapple Quality. (XLSX)
